# Impact of visual discomfort symptoms on SDMT performance among persons with MS

**DOI:** 10.3389/fneur.2025.1569451

**Published:** 2025-06-16

**Authors:** Michelle H. Chen, Timothy J. Rich, Nancy Chiaravalloti, Yael Goverover, Silvana L. Costa

**Affiliations:** ^1^Institute for Health, Health Care Policy and Aging Research, Rutgers University, New Brunswick, NJ, United States; ^2^Department of Neurology, Robert Wood Johnson Medical School, Rutgers University, New Brunswick, NJ, United States; ^3^Center for Stroke Rehabilitation Research, Kessler Foundation, East Hanover, West Orange, NJ, United States; ^4^Department of Physical Medicine and Rehabilitation, New Jersey Medical School, Rutgers University, Newark, NJ, United States; ^5^Center for Neuropsychology and Neuroscience, Kessler Foundation, East Hanover, NJ, United States; ^6^Department of Occupational Therapy, Steinhardt School of Culture, Education, and Human Development, New York University, NY, United States

**Keywords:** visual impairment, visual discomfort scale, symbol digit modalities test, information processing speed, multiple sclerosis

## Abstract

**Background:**

Visual problems are common among persons with multiple sclerosis (MS) and may interfere with the assessment of cognitive functioning using visually mediated neuropsychological tests. The current study explored visual discomfort symptoms among persons with MS compared to healthy controls (HCs), using the Visual Discomfort Scale (VDS), which measures somatic and perceptual visual discomfort symptoms that interfere with reading.

**Methods:**

Eighty-nine persons with MS and 30 HCs completed the VDS and the Symbol Digit Modalities Test (SDMT), a visually mediated test of information processing speed and gold standard for screening for MS-related cognitive dysfunction.

**Results:**

Persons with MS endorsed higher frequencies of visual discomfort symptoms, including seeing the text or background moving or fading, headache/eye soreness, blurriness/diplopia, having to re-read, and slow reading, compared to HCs. More frequent visual discomfort symptoms were associated with worse performance on the SDMT. For participants with MS reporting moderate/high levels of visual discomfort symptoms, having a longer disease duration or progressive disease courses were correlated with worse performance on the SDMT.

**Conclusion:**

It is important for clinicians to ask about specific visual discomfort problems that the patient experiences when interpreting a visually-mediated neuropsychological test such as the SDMT, especially for MS patients with longer disease duration or a progressive disease course.

## Introduction

Multiple sclerosis (MS) is a demyelinating, inflammatory, and neurodegenerative condition, affecting 2.2 million individuals globally (1). Demyelination and inflammation of the optic nerve (optic neuritis) is one of the most common presenting symptoms of MS (2). Although optic neuritis is transient, many persons with MS report persistent visual problems even after resolution of optic neuritis (3). Using structural imaging techniques, such as optical coherence tomography (OCT) and magnetic resonance imaging (MRI), we now know that there is neurodegeneration in the visual pathway over time among persons with MS in addition to the acute neuro-ophthalmic episodes (4).

Visual impairment significantly disrupts health-related quality of life among persons with MS (5, 6). Most commonly reported problems include blurry or double vision, trouble seeing at night or under bright sunlight, difficulty with reading or looking at a computer, and driving or parking a car (5, 6). The 25-Item National Eye Institute Visual Function Questionnaire (NEI-VFQ-25) is the most widely used vision-related patient reported outcome measure in MS. The NEI-VFQ-25 composite and subscale scores have been validated in the MS population, including discriminating between MS and healthy control (HC) participants as well as correlating with performance on visual function tests, such as visual acuity and low contrast sensitivity (5–8). A 10-item Neuro-Ophthalmic Supplement to the NEI-VFQ-25 was developed to increase the measure’s sensitivity in the detection of neuro-ophthalmologic symptoms, including blurry vision, trouble following moving objects, and double vision (9).

While the NEI-VFQ-25 is a well validated measure that effectively represents the real-life impact of visual impairment, its emphasis is on gauging the *degree* to which visual symptoms disrupt daily activities rather than the *manner* in which they are disrupted. Visual Discomfort Scale (VDS) (10, 11)explores in greater detail the somatic and perceptual symptoms experienced by those with visual discomfort that interfere with reading and similar tasks. The VDS is a 23-item measure that probes physical symptoms such as strained eyes and headache; perceptual symptoms such as moving, floating, or flickering text; and compensatory strategies employed such as squinting, repetitive blinking, or using one’s finger to guide the eyes across the text. The VDS can therefore provide more clinically relevant information that may direct intervention to reduce somatic and perceptual symptoms of visual stress during reading that the NEI-VFQ-25 cannot.

Cognitive impairment is also prevalent and debilitating at onset and throughout the course of MS (12). Although variability in cognitive impairment has been reported (13, 14), it occurs across all MS types with prevalence in the range of 25–75% of the overall MS population (13, 15). It most often affects processing speed, executive functioning, visual and verbal memory, and visuospatial processing (13). Furthermore, cognitive impairment may predate other symptoms of MS (13, 14). Cognitive dysfunction has been shown to negatively impact quality of life, including employment and social functioning (15–18) [cf. Chow et al. (19), Glanz et al. (20), and Baumstarck-Barrau et al. (21)].

Tests used to assess the most commonly affected cognitive domains in MS, such as information processing speed, visual memory, and executive functions, are often exclusively visually-mediated. It is thus necessary to examine how visual problems may confound performance on these neuropsychological tests. Accurate detection of cognitive deficits is vital in monitoring disease progression and treatment planning.

We have previously shown that a history of neuro-ophthalmic syndromes and oculomotor speed deficits are associated with poorer performance on the Symbol Digit Modalities Test (SDMT) (22, 23), a visually-mediated measure of information processing speed and a gold standard screening measure for MS-related cognitive dysfunction (24). Other studies have also found that those who performed worse on visual function tests also performed poorly on visually-mediated neuropsychological tests (25–27). The current study will extend these findings by examining if self-reported visual discomfort problems would also be associated with SDMT performance.

The overarching objective of the current investigation is to explore which visual discomfort problems are commonly reported among persons with MS and whether they are discrepant from HCs. We hypothesize that persons with MS would report more frequent visual discomfort symptoms compared to HCs. Moreover, the current study will examine whether self-reported visual discomfort symptoms are associated with performance on the SDMT. We hypothesize that more frequent visual discomfort symptoms would be associated with poorer performance on the SDMT. Finally, we will evaluate the role of MS disease characteristics in visual discomfort symptoms and their association with SDMT performance. We hypothesize that longer disease duration and progressive MS disease course would be linked to more frequent visual discomfort problems.

## Methods

### Participants

Data from four studies were included in the current investigation. Inclusion and exclusion criteria were similar across studies. Inclusion criteria included: (1) diagnosis of MS (or no neurological conditions for the HC group), (2) able to speak English fluently, and (3) age between 18 and 70 years. Exclusion criteria included: (1) history of stroke or neurologic disease other than MS, (2) history of significant psychiatric disorders (e.g., major depressive disorder, bipolar disorder, schizophrenia), (3) history of alcohol or substance abuse, (4) MS relapses within the past month, and (5) use of medications that may influence cognition, such as steroids, benzodiazepines, neuroleptics, and opiates, within the past month. Clinical variables such as, but not limited to, time since diagnosis, relapsed history, use of disease modifying therapies, were retrieved from medical records, when available, or by participant self-report. All studies were approved by the Kessler Foundation Institutional Review Board, and all participants provided written informed consent before enrollment.

### Measures

The SDMT assesses information processing speed and is a gold standard for evaluating cognition in MS research (28, 29). The oral version of the SDMT was used for this study, as recommended in MS consensus neuropsychological batteries (30, 31), due to motor difficulties among MS participants that may confound performance on the written version. Participants were presented with an 8.5 × 11 sheet of paper with a key of nine symbols and digit pairs along the top. Below the key were several lines of symbols without the corresponding number. The participant was asked to verbally call out numbers for different symbols as quickly as they could within 90 s, after completing several practice trials. The total number of correct responses was used as an outcome for this study.

The VDS is 23-item questionnaire assessing the frequency of visual discomfort symptoms (10, 11). Responses were recorded in a four-point Likert scale, from “never occurs” to “almost always.” Higher scores indicated more frequent visual discomfort symptoms. Besides a total score, we also calculated scores for each domain, including seeing the text or background moving or fading, headache/eye soreness, blurriness/diplopia, having to re-read, experience of glare, and slow reading, based on factor analyses performed in prior literature (11). The total score was further divided into three clinical ranges based on previous literature (low = 0–24; moderate = 25–48; high = 49–69) (10, 11).

### Statistical analyses

R version 4.0.4 was used for all analyses. Group differences (MS vs. HC) in demographic characteristics were determined using Welch’s Two Samples t tests for continuous variables and Pearson’s chi-squared tests for categorical variables. Group differences in VDS scores were calculated using generalized linear models with Poisson distributions, adjusted for age. For VDS clinical ranges, because only one person was in the high visual discomfort group, they were combined with the moderate group for subsequent analyses; group differences in clinical ranges were determined using a generalized linear model with binomial distribution (logistic). Relationships between MS disease variables (duration and disease courses) and VDS scores were examined using Poisson generalized linear models (binomial models for clinical ranges), adjusted for age. In this analysis, MS disease course was dichotomized into relapsing–remitting or progressive types. Associations between VDS scores and the SDMT score were calculated using Poisson generalized linear models, adjusted for both age and education, within the overall sample (MS and HC). Potential moderating effects of MS disease variables on the relationship between VDS and SDMT scores were investigated with additional MS disease × VDS interaction terms. Predictors were centered on reducing the correlation between the interaction terms and their component predictors.

## Results

### Demographic and clinical characteristics

The study sample included 89 participants with MS and 30 healthy controls (HCs). See Table 1 for summary of demographic and disease characteristics. The MS group was significantly older [*t* (46.09) = −4.03, *p* < 0.001] and had more females than the HC group [*χ*^2^(1) = 5.14, *p* = 0.023].

**Table 1:** Demographic and disease characteristics of the sample.

Demographic or disease characteristic	MS (*n* = 89)		HC (*n* = 30)		MS vs. HC
	Mean (SD)	Range	Mean (SD)	Range	*t*/*χ*^2^, *P*
Age: years	52.83 (9.39)	33–70	44.2 (10.37)	24–62	−4.04, <0.001
Education: years	15.9 (2.5)	12–24	16.2 (2.35)	12–23	0.60, 0.553
MS disease duration: years	12.4 (11.55)	3 months – 41 years	N/A	N/A	N/A
	Number (%)		Number (%)		
Female	62 (69.7)		14 (46.7)		5.14, 0.023
MS disease course					N/A
Relapsing–remitting	54 (60.7)		N/A		
Primary progressive	6 (6.7)		N/A		
Secondary progressive	18 (20.2)		N/A		
Unknown	11 (12.4)				

Group differences (MS vs. HC) were tested with Welch’s *t* test and categorical variables were evaluated using Pearson’s chi-squared test. MS: multiple sclerosis. HC: healthy control. SD: standard deviation.

### Group differences on the VDS

The MS group endorsed more frequent visual discomfort symptoms on the VDS on all domains except for experience of glare (Table 2). Using Poisson generalized linear models adjusted for age, the group effects were: estimate = 1.45, *p* < 0.001 for total score; estimate = 3.14, *p* < 0.001 for movement/fading; estimate = 0.57, *p* = 0.004 for headache/soreness; estimate = 2.23, *p* < 0.001 for blur/diplopia; estimate = 1.04, *p* < 0.001 for rereading; estimate = 0.27, *p* = 0.513 for glare; estimate = 3.05, *p* = 0.003 for slow reading. All HCs and the majority of persons with MS were in the low visual discomfort group, but 20% of persons with MS fell in the moderate visual discomfort group and one person with MS fell in the high visual discomfort group. However, formal testing of group differences was not significant (*p* > 0.05).

**Table 2:** VDS scores.

VDS score	MS (*n* = 89)		HC (*n* = 30)		MS vs. HC
	Median (IQR)	Range	Median (IQR)	Range	estimate, *P*
VDS total score	13 (15)	0–55	4 (4)	0–12	1.45, <0.001
VDS movement/fading subscore	1 (4)	0–17	0 (0)	0–3	3.14, <0.001
VDS headache/soreness subscore	3 (4)	0–12	2 (2.5)	0–6	0.57, 0.004
VDS blur/diplopia subscore	2.5 (3)	0–11	0 (0.5)	0–3	2.23, <0.001
VDS rereading subscore	4 (4)	0–9	1 (1.5)	0–6	1.04, <0.001
VDS glare subscore	1 (1)	0–3	1 (1)	0–1	0.27, 0.513
VDS slow reading subscore	1 (2)	0–3	0 (0)	0–1	3.05, 0.002
VDS total score range	Number (%)		Number (%)		18.70, 0.992
Low	70 (78.7)		30 (100)		
Moderate	18 (20.2)		0 (0)		
High	1 (1.1)		0 (0)		

Group differences were tested by generalized linear models adjusted for age, with Poisson distributions for continuous VDS scores and binomial distributions (logistic) VDS categorical ranges. VDS, visual discomfort scale; MS, multiple sclerosis; HC, healthy control; IQR, interquartile range.

### Associations between visual discomfort symptoms and SDMT performance

**Figure 1:**
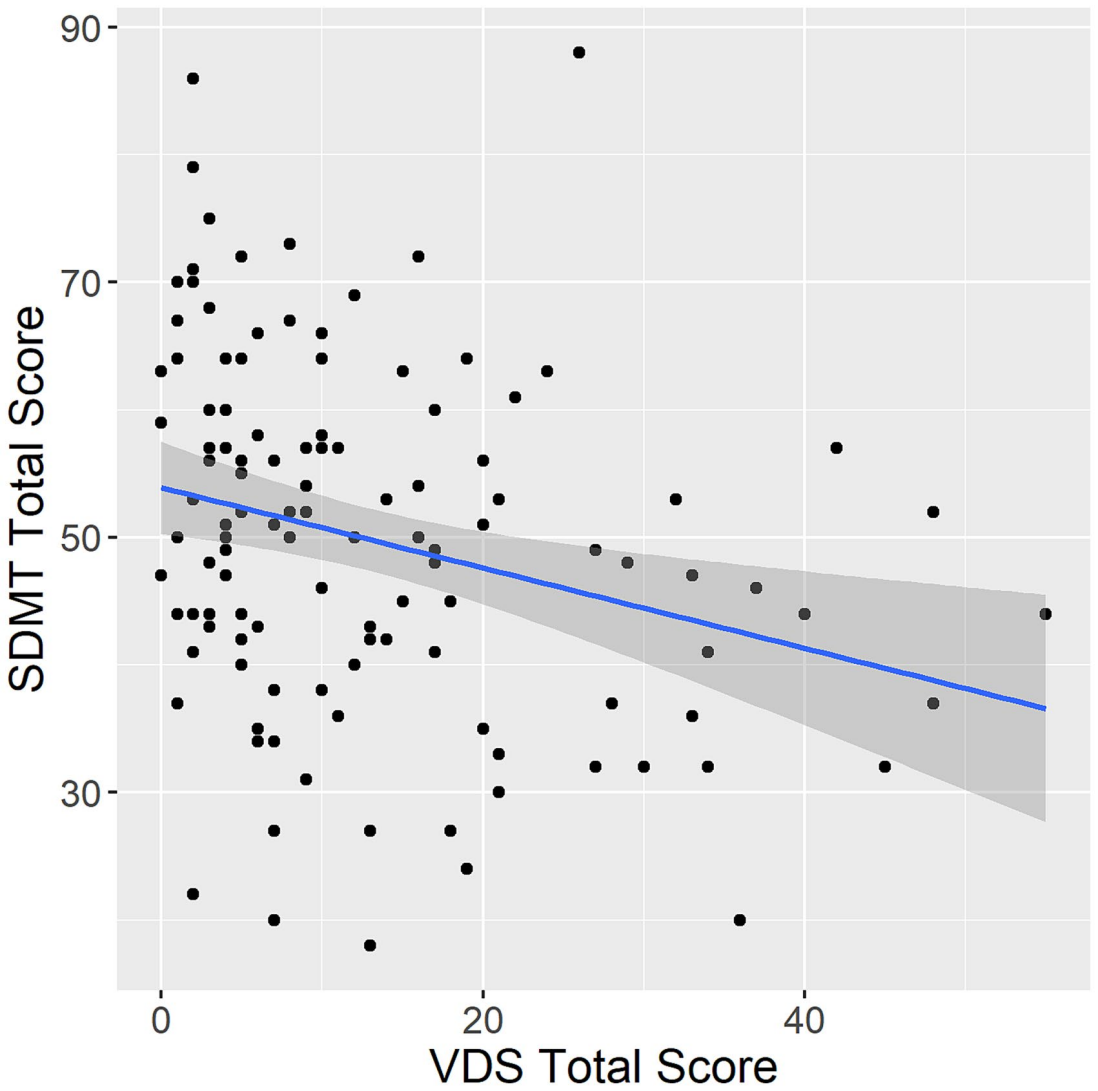
Association between VDS and SDMT. VDS, visual discomfort scale. SDMT, symbol digit modalities test. Error band represents 95% confidence intervals.

In Poisson generalized linear models adjusted for both age and education within the overall sample, higher frequency in most visual discomfort symptoms was associated with poorer performance on the SDMT. Specifically, all but headache/soreness and glare symptom scores were associated with performance on the SDMT (total score: estimate = −5.94 × 10^−3^, *p* < 0.001; movement/fading: estimate = −0.02, *p* < 0.001; headache/soreness: estimate = −5.21 × 10^−3^, *p* = 0.330; blur/diplopia: estimate = −0.01, *p* = 0.012; rereading: estimate = −0.03, *p* < 0.001; glare: estimate = −0.03, *p* = 0.160; slow reading: estimate = −0.07, *p* < 0.001; Figure 1). Participants in the low visual discomfort group had higher SDMT scores than participants in the moderate or high visual discomfort group (estimate = −0.14, *p* < 0.001).

### Role of MS disease characteristics

MS disease duration was associated with only the headache/soreness symptom (estimate = 0.001, *p* = 0.042) but not VDS total score or any other visual discomfort symptoms, after adjusting for age. Interestingly, after adjusting for age, MS participants with the relapsing–remitting disease course endorsed more frequent visual discomfort symptoms compared to those with the progressive disease course in the VDS total score (estimate = 0.17, *p* = 0.004). However, this association was not maintained with individual symptom scores (*p* > 0.05). Neither MS disease duration nor disease course was associated with VDS clinical ranges (*p* > 0.05).

With regard to the role of MS disease variables in moderating the relationships between VDS and SDMT scores, MS participants with longer disease duration exhibited a stronger negative correlation between VDS total score and SDMT score (interaction estimate = −2.88 × 10^−5^, *p* = 0.019), adjusting for age and education. The same moderating effect was observed in the blur/diplopia (interaction estimate = −1.66 × 10^−4^, *p* = 0.001), glare (interaction estimate = −3.05 × 10^−4^, *p* = 0.042), and slow reading (interaction estimate = −2.95 × 10^−4^, *p* = 0.015) symptom scores. Having a progressive course yielded a stronger negative correlation between visual discomfort symptoms and SDMT performance only for the glare symptom (interaction estimate = 0.09, *p* = 0.029), adjusting for age and education.

**Figure 2:**
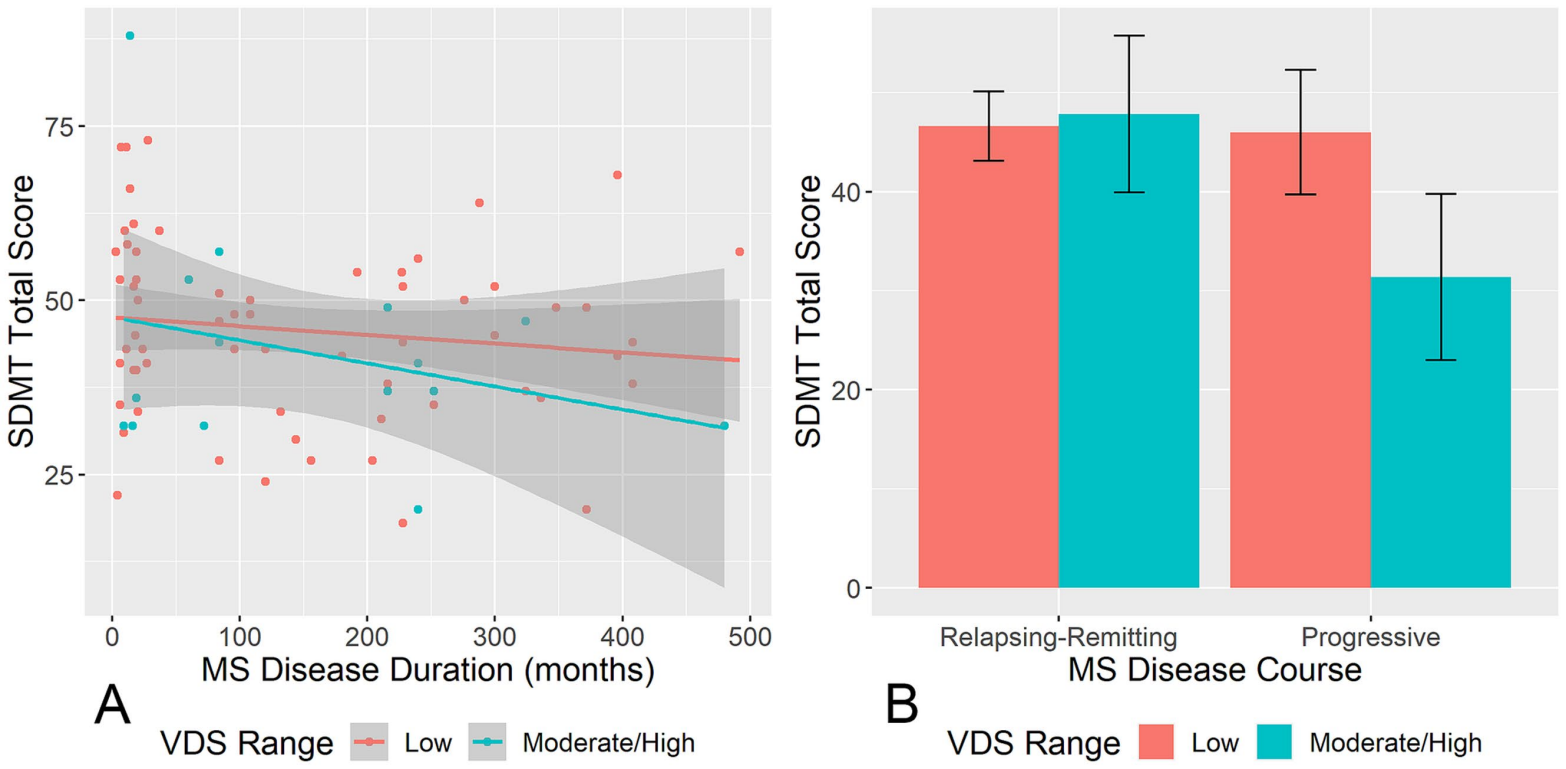
Moderating effects of MS disease characteristics on the relationships between VDS and SDMT. **(A)** Shows the moderating effect of VDS range on the relationship between MS disease duration and SDMT score. **(B)** Shows the moderating effect of VDS range on the relationship between MS disease course and SDMT score. VDS, visual discomfort scale; SDMT, symbol digit modalities test. Error bands/bars represent 95% confidence intervals.

Notably, the negative correlation between MS disease duration and SDMT score was stronger among participants in the moderate/high visual discomfort group relative to the low visual discomfort group (interaction estimate = 7.03 × 10^−4^, *p* = 0.04; Figure 2A), adjusting for age and education. MS participants with a progressive course in the moderate/high visual discomfort group had lower SDMT score than those with a progressive course in the low visual discomfort group or MS participants with relapsing–remitting course in either low or high visual discomfort group (interaction estimate = 0.34, *p* < 0.001; Figure 2B).

## Discussion

The current study was the first to examine somatic and perceptual visual discomfort problems using the VDS in persons with MS. Across a variety of domains, persons with MS endorsed higher frequencies of visual discomfort symptoms relative to HCs. More frequent visual discomfort symptoms were associated with worse performance on the SDMT. For participants with MS reporting moderate/high levels of visual discomfort symptoms, having a longer disease duration or progressive disease courses (primary or secondary) were linked with worse performance on the SDMT.

The current study extends previous vision research in MS. While studies that use neuroimaging or visual/oculomotor assessment as outcome measures elucidate the neurodegenerative processes that affect vision and ocular motility, they do not capture the subjective experience of visual discomfort and its impact on daily living. Clinicians rely on data derived from both objective and subjective measurement to implement targeted, evidence-based interventions customized to the unique needs of each patient. The most widely used patient-reported outcome measure in MS research, the NEI-VFQ-25 (32), measures the degree of interference of visual problems on various activities of daily living but is not specific to the type of visual problem that is occurring. The emphasis of the VDS, on the other hand, is on the individual experience of somatic and perceptual symptoms that typically accompany visual stress. Therefore, the VDS may better inform treatment than the NEI-VFQ-25.

Our finding that more frequent visual discomfort symptoms were associated with worse performance on the SDMT is consistent with our prior investigation, which found that visual and oculomotor deficits were related to worse SDMT performance (22). Similarly, other studies have found that poor performance on visual function tests (e.g., visual acuity, and low-contrast sensitivity) was linked to worse performance on visually-mediated neuropsychological tests among persons with MS (16–18, 25–27). Importantly, specific symptoms that were related to SDMT performance included seeing the text or background moving or fading, blurriness/diplopia, having to re-read, and reading slowly. In contrast, headache/eye soreness and experience of glare did not impact SDMT performance. Additional evidence of interaction between cognitive and visual impairments has been provided by studies using Optical Coherence Tomography (OCT). OCT metrics, such as, retinal nerve fiber layer thickness, have been proven to be a sensitive biomarker of neurodegeneration in MS (33–35) and is highly correlated with performance on cognitive tests, such as the SDMT (36, 37). Thus, it is important for clinicians to objectively and subjectively assess visual discomfort problems that the patient experiences when interpreting a visually-mediated neuropsychological test such as the SDMT, especially among those with a longer disease duration or progressive disease course. The VDS is quick to administer in a clinical setting, with 23 items using a four-point Likert scale.

Additionally, we found that having a longer disease duration and progressive disease course were associated with worse performance on the SDMT only among those with a clinically moderate or high level of visual discomfort (relative to a low level). This suggests that poor performance observed on the SDMT among patients with longer disease duration and progressive disease course may be partially due to their visual problems, rather than purely cognitive dysfunction. It is well known that age and lifestyle factors, such as diet, can influence both cognitive and visual outcomes (38, 39); future research should extend the current research by exploring the multifaceted processes that influence both cognitive and visual outcomes.

There were a few limitations to this study that warrant discussion. First, the cross-sectional nature of the current investigation precludes us from making statements regarding causation. While it is tempting to conclude that visual discomfort symptoms were a confounder of SDMT performance, it is also possible that MS-related neurodegeneration manifests in both visual discomfort and cognitive deficits. More longitudinal research examining vision and cognition, with neuroimaging measures, are needed to disentangle these cause-and-effect relationships. Second, we used groups of unequal size (30 healthy controls vs. 89 MS patients). Finally, the VDS has not been formally validated in the MS population, and the current study lacked some important clinical measures, such as objective visual function tests and neurologic disability measures (e.g., Kurtzke Expanded Disability Status Scale). Future research should include these measures which will add to our understanding of visual discomfort symptoms among persons with MS.

In conclusion, MS patients with greater self-report of symptoms of visual discomfort showed worse performance on the SDMT. This could reflect that MS-related neurodegeneration manifests in both visual discomfort and cognitive impairment, or that performance on visually-mediated neuropsychological tests such as the SDMT is mediated by symptoms of visual discomfort. Thus, clinicians should identify specific visual discomfort problems that the patient experiences and take them into account when interpreting a visually-mediated neuropsychological test such as the SDMT, especially for MS patients with longer disease duration or a progressive disease course.

## Data Availability

The raw data supporting the conclusions of this article will be made available by the authors, without undue reservation.
